# Correlation between Hyperhomocysteinemia and Common Carotid Artery Intima media Thickness in Carbamazepine treated Epileptic patients using Ultrasonography

**DOI:** 10.12669/pjms.335.12982

**Published:** 2017

**Authors:** Shazia Bano, Nudrat Anwar Zuberi, Syed Munawar Alam

**Affiliations:** 1 Dr. Shazia Bano, (MBBS, M.Phil), Assistant Professor, Department of Biochemistry, Bolan Medical College, Quetta, Pakistan; 2 Prof. Dr. Nudrat Anwar Zuberi,(M.Phil, PhD), Professor, Department of Biochemistry; 3 Unaizah College of Medicine, Qassim University, Saudi Arabia; 4 Dr. Syed Munawar Alam, (MBBS, M.Phil), Assistant Professor, Department of Biochemistry, Fatima Jinnah Dental College, Karachi, Pakistan

**Keywords:** Endothelial dysfunction, Epilepsy, Homocysteine, Monotherapy, Seizures

## Abstract

**Objective:**

The objective was to assess the role of homocysteine in the development of atherosclerosis in common carotid artery in the carbamazepine treated epileptic patients.

**Methods:**

This study was conducted in the Department of Biochemistry, Basic Medical Sciences Institute (BMSI), Jinnah Postgraduate Medical Center (JPMC), Karachi. Three hundred individuals, aged 34 ± 9.5 years were selected and divided into three groups. Each group comprised of 100 subjects labeled as Group-A (control group had healthy individuals), Group-B (newly diagnosed epileptic patients without antiepileptic therapy), Group-C (epileptic patients on Carbamazepine therapy, which was further subdivided into C-I having epileptic patients on Carbamazepine therapy less than 1 year n=33, C-II had epileptic patients on Carbamazepine therapy 1-2 years n = 33 and C-III comprised of epileptic patients on Carbamazepine therapy more than 2 years n = 34). Blood concentration of homocysteine was measured and ultrasound of Common Carotid Artery for intima-media thickness was performed.

**Results:**

Significantly elevated level of homocysteine was observed in epileptic patients on CBZ therapy. Common Carotid Artery Intima-media thickness (CCA IMT) was observed significantly high throughout group C but it was more profound in Group-C-III. Homocysteine was found positively correlated with right CCA IMT, left CCA IMT and mean CCA IMT.

**Conclusion:**

Hyperhomocysteinemia was linked with increased risk of atherosclerosis in CBZ treated epileptic patients.

## INTRODUCTION

Epilepsy is a chronic neurological disorder characterized by recurrent seizures, which are caused by excessive discharges in the neurons.^1^,^2^ Epilepsy has no relation with age, race, socio-geographic or national boundaries.^3^ Different drugs for the treatment of epilepsy are available but the most common treatment for epilepsy is monotherapy with antiepileptic drugs (AEDs) and about 50% patients are treated successfully with monotherapy.^4^ Carbamazepine (CBZ) is used as a first line drug to control different forms of epilepsy. It is one of the first AEDs introduced in 1953 by a known pharmaceutical company.^5^ Many studies have suggested that the plasma homocysteine (Hcy) level is increased as a side effect with prolong use of some AEDs, which is associated with vascular endothelial injury; hence it increases the risk of atherosclerosis.^6^,^7^

Carbamazepine is an enzyme inducing drug; it causes deficiency of cofactors of Hcy metabolism and leads to change in Hcy level. Homocysteine is a sulfur containing amino acid which is formed during the metabolism of methionine.^8^ The one half of Hcy is used in synthesis of cysteine via trans-sulfuration by vitamin B6 dependent enzyme, cystathionine beta synthase (CBS). The other half of Hcy is remethylated to methionine, with cofactors like folic acid and vitamin B_12_. In the deficiency of these vitamins the Hcy level is increased, which is a well-established risk factor for vascular endothelial injury resulting in increased risk of atherosclerosis.^9^

Hyperhomocysteinemia is defined as Hcy level above 15 µmol/l,^10^,^11^ which is moderate (16-30 µmol/l), intermediate (31-100 µmol/l) and severe (>100 µmol/l).^10^ It is suggested that high level of Hcy causes injury to epithelial cells by bringing changes in pro-coagulant activity, platelet activity and in LDL-C.^12^ Excessive production of reactive oxygen species due to Hyperhomocysteinemia hyperhomocystenemiaaffects the cellular antioxidants which brings oxidative changes in LDL-C and causes inflammatory changes which lead to formation of atherosclerotic plug.^13^ Common Carotid Artery intima-media thickness (CCA IMT) is an important determinant of atherosclerosis.^6^

The objective was to assess the role of homocysteine in the development of atherosclerosis in common carotid artery in the carbamazepine treated epileptic patients.

## METHODS

A cross-sectional study was performed in the department of Biochemistry, Basic Medical Sciences Institute, Jinnah Post Graduate Medical Centre Karachi, from January 2015 to January 2016. The sample size was calculated by open epi calculator, using Bhesania et al., 2014 as a reference study. Three hundred individuals, aged 34 ± 9.5 years were selected (Total 200 cases from Neurology Department and National Epilepsy Centre of JPMC and 100 healthy subjects from healthy population were included in the study after fulfilling the inclusion criteria). For the recruitment of healthy subjects/controls; demographically matched healthy individuals without any history of epilepsy were approached, they were briefed about the context of participation in the study and after having their informed consent in black and white, they were screened as per the inclusion/exclusion criteria and only those subjects/volunteers who fulfil the prerequisite were included as control in the study. As per the gender distribution of these participants more than 54% (n=163) were male and the rest of around 46% (n=137) were female. The mean BMI of the study participants was 25 ± 1.88 therefore it would be safe to transcribe that they were borderline obese as per the anthropometric classifications for Asian habitants. The study participants were then divided into three groups. Each group comprised of 100 subjects labeled as Group-A (control group had healthy individuals), Group-B (newly diagnosed epileptic patients without antiepileptic therapy), Group-C (epileptic patients on Carbamazepine therapy, which was further subdivided into C-I having epileptic patients on Carbamazepine therapy less than 1 year n = 33, C-II had epileptic patients on Carbamazepine therapy 1-2 years n = 33 and C-III comprised of epileptic patients on Carbamazepine therapy more than 2 years n = 34). Either newly diagnosed cases of generalized epilepsy without antiepileptic treatment or known cases of generalized epilepsy on carbamazepine monotherapy were included in the study along with the age and gender matched healthy subjects as controls. Epileptic patients having following comorbidities and habits were excluded: known cases of ischemic heart diseases, renal diseases, liver diseases, hypertension, autoimmune disease, tobacco smokers, pregnant and lactating women. An ethical approval was taken from the Institutional Review Board of BMSI, JPMC, Karachi as well as from the Ethical Committee of Jinnah Post Graduate Medical Centre Karachi. The written informed consent was taken from every subject. Serum homocysteine level was estimated by ELISA Kit, manufactured by SHANGHAI YEHUA Biological Technology Co., Ltd.

Assessment of atherosclerosis was done by measuring CCA IMT by ultrasonography, in the Radiology Department of JPMC (Doppler Ultra Sound machine, model AY-15CUI, Korea). Images were taken from B-mode ultrasound system, equipped with linear probe of 7.5 MHz. Subjects were in supine position. Neck was turned to left for the measurement of right CCA IMT and to right for the measurement of left CCA IMT. Common carotid artery intima-media thickness was measured proximal to the 1cm of bifurcation of CCA. Thickness between the intima and media-adventitia was measured for both sides and mean CCA IMT was calculated.

SPSS version 16 was used for data analysis. Data was entered as mean ± SD. One -way Analysis of Variance (ANOVA) and post hoc Tukey was used for the comparison of means among more than two variables. The Pearson correlation was applied to correlate the levels of serum homocysteine with other study variables. The statistical tests were considered significant at p value < 0.05.

## RESULTS

### Comparison of Homocysteine among study groups

The first evident finding in our study was a statistically significant variation in the mean homocysteine level among the study groups. The p-value was found statistically significant when homocysteine was compared among the five study groups (p-value = 0.001).

The post hoc analysis (Table-I) showed that the mean homocysteine (µmol/l) level was significantly different between Group-A and Group-C-I (11.2 ± 2.24 vs. 16.25 ± 1.33; p-value = 0.001).

**Table-I:** Comparison of Homocysteine and Common Carotid Artery Intima-Media Thickness among Study Groups.

*Variables*	*Group-A*	*Group-B*	*Group-C-I*	*Group-C-II*	*Group-C-III*
Homocysteine (µmol/l)	11.2 ± 2.24	10.18 ± 2.08	*† 16.25 ± 1.33	*†♦ 23.58 ± 3.2	*†♦§ 31.41 ± 5.41
Right CCA IMT (mm)	0.46 ± 0.07	0.47 ± 0.08	0.47 ± 0.07	0.48 ± 0.07	*†♦§ 0.59 ± 0.15
Left CCA IMT (mm)	0.46 ± 0.06	0.47 ± 0.07	0.44 ± 0.07	0.45 ± 0.05	*†♦§ 0.56 ± 0.13
Mean CCA IMT (mm)	0.46 ± 0.04	0.47 ± 0.05	0.46 ± 0.05	0.46 ± 0.04	*†♦§ 0.57 ± 0.09

Values were expressed as mean ± SD;

* statistically significant as compared to Group-A

† statistically significant as compared to Group-B;

♦ statistically significant as compared to Group-C-I

§ Statistically significant as compared to Group-C-II; (p value < 0.05 is statistically significant).

### Comparison of Common Carotid Artery intima-media thickness among study groups

As per our hypothesis, it was also a potentially anticipated finding that the mean intima media thickness of common carotid arteries was also different and statistically significant among the study groups.

### Correlation of Homocysteine (µmol/l) with Common Carotid Artery intima media thickness (mm)

When bivariate Pearson correlation was conducted between the predictor variable (serum homocysteine) and outcome variable (Common carotid artery intima media thickness) (Table-II), all three; Right CCA IMT, Left CCA IMT and Mean CCA IMT were found significantly and positively correlated with homocysteine at p-value < 0.01 (Fig.I).

**Table-II:** Correlation of Homocysteine (µmol/l) with Common Carotid Artery Intima-Media Thickness (mm).

*Variables*	*Correlation Co-efficient (r)*
Right CCA IMT (mm)	0.354**
Left CCA IMT (mm)	0.227**
Mean CCA IMT (mm)	0.419**

** Statistically significant < 0.01.

**Fig.I:**
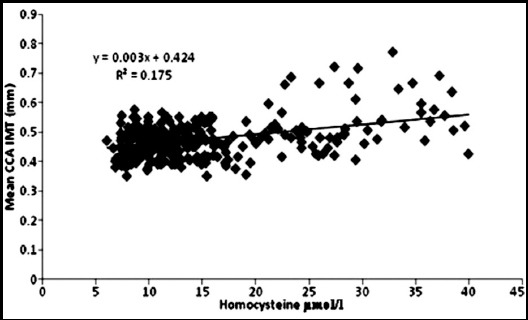
Correlation of Homocysteine (µmol/l) with Mean CCA IMT (mm).

### Comparison of different levels of Homocysteine with different durations of Carbamazepine Therapy

The cross tabulation (Table III) was performed between different levels of homocysteine and duration of Carbamazepine therapy. Greater proportion of patients with serum homocysteine > 30 µmol/l were in the epileptic patients on Carbamazepine therapy > 2 years group. There was significant difference found among the proportions with p-value < 0.001.

**Table-III:** Comparison of different levels of Homocysteine with different durations of Carbamazepine Therapy.

	*Group C-I*	*Group C-II*	*Group C-III*	*p-value*
Serum homocysteine (<16 µmol/l)	15 (45.5)	0 (0)	0 (0)	0.001
Serum homocysteine (16-30 µmol/l)	18 (54.5)	33 (100)	14 (41.2)	
Serum homocysteine (> 30 µmol/l)	0 (0)	0 (0)	20 (58.8)	

Values were expressed as frequency and percentage. Statistically significant difference was found among the groups compared (p value < 0.01).

## DISCUSSION

Homocysteine is a non-essential amino acid and vascular marker. Prolong use of enzyme-inducing AEDs significantly increases Hcy level.^14^,^15^ High serum levels of Hcy are associated with increased mortality rate due to ischemic heart diseases. It is demonstrated that the risk of cardiovascular diseases increases up to 20% by an increase of 5mmol/l in plasma homocysteine level.^11^

Increased Hcy concentration causes increased production of reactive oxygen species, increased platelets aggregation and increases the release of inflammatory mediators and inhibits protein C. All of these factors cause prothrombotic activity of Hcy.^16^,^17^

We have found that level of Hcy was significantly increased with the increase in duration of CBZ therapy. In concurrence to our results Verrotti et al.^18^ and Karabiber et al.^19^ have reported significant increase in Hcy level in epileptic patients after 1 year of CBZ therapy.

Chuang et al.^17^ and El-Farahaty et al.^20^ have reported significant increase in Hcy level in epileptic patients after 2 years of CBZ therapy, while in conflict to our results Kumar et al.^21^ reported insignificant change in the level of Hcy after six months of therapy and Abd El Dayem et al.^22^ after one year of therapy.

We have not found significant difference in Hcy level between controls and newly diagnosed epileptic patients without treatment. A study done by Eldeen et al.^8^ suggested that increased level of Hcy may be due to AEDs when there is no significant difference between control and non AED users. Antiepileptic drugs cause hyperhomocysteinemia due to decreased absorption and increased metabolism of vitamin B12 and folate.^8^

We have found significant positive correlation of homocysteine with duration of CBZ therapy at 95% confidence interval which is in accordance with findings of Eldeen et al.^8^ Duration of AED is considerably linked with increase of rate of atherosclerosis in epileptic patients.^6^ The significant results were obtained on ultrasound. We have found significant increased right CCA IMT, left CCA IMT and mean CCA IMT in epileptic patients on CBZ therapy more than 2 years and Hcy has significant positive correlation with right CCA IMT, left CCA IMT and mean CCA IMT. Chuang et al.,^13^ have reported significant increased right CCA IMT and mean CCA IMT; however Tan et al.,^6^ have found insignificant correlation of Hcy with CCA IMT.

The study have some limitations, due to the cross sectional study design, different subjects for “newly diagnosed epileptic patients” and “epileptic patients on different durations of CBZ therapy” were recruited in the study rather than opting for the selection and recruitment of the same subjects who could be followed over longitudinally against the baseline indicators. As it was difficult to design and execute the follow up due to the large number of patients especially in a public sector teaching hospital, funding problems and time limitations, we were apprehending a large percentage of loss to follow up subjects in that scenario therefore the cross sectional study design was planned. Although we have recruited 300 subjects, still the groups comprised on epileptic patients on different durations of CBZ therapy have small number of patients. This may not be enough to reflect the better estimate of atherosclerosis.

It is recommended that Homocysteine should be considered as a potential risk factor for atherosclerotic changes in CBZ treated epileptic patients. It should be advised to avoid high cholesterol diet in epileptic patients on Carbamazepine therapy and these patients should be routinely monitored for homocysteine level to check the progression of atherosclerotic changes.

## CONCLUSION

Our study results indicated that homocysteine may have a role in the development of atherosclerotic changes in epileptic patients on Carbamazepine therapy and it may be an early predictor to assess the risk of atherosclerosis in these patients.

### Authors` Contribution

**SB:** Designed the study, collected the samples and prepared the manuscript.

**NAZ:** Supervised the study, and helped in drafting and revising the manuscript.

**SMA:** Helped in drafting, revising and finalizing the manuscript.

The final manuscript was approved by all authors for publication.
